# Biosynthesis of Ascorbic Acid as a Glucose-Induced Photoprotective Process in the Extremophilic Red Alga *Galdieria partita*

**DOI:** 10.3389/fmicb.2019.03005

**Published:** 2020-01-14

**Authors:** Han-Yi Fu, Shao-Lun Liu, Yin-Ru Chiang

**Affiliations:** ^1^Department of Biological Sciences, National Sun Yat-sen University, Kaohsiung, Taiwan; ^2^Department of Life Science and Center for Ecology and Environment, Tunghai University, Taichung, Taiwan; ^3^Biodiversity Research Center, Academia Sinica, Taipei, Taiwan

**Keywords:** acidothermophilic red algae, reactive oxygen species, P700, heterotrophy, mixotrophy

## Abstract

The extremophilic red alga *Galdieria partita* is a facultative heterotroph that occupies mostly low-light microhabitats. However, the exceptional detection of abundant populations of *G. partita* in sunlight-exposed soil raises the possibility that exogenous organic carbon sources protect cells from photo-oxidative damage. The present study aimed to identify the photoprotective process activated by exogenous glucose under photo-oxidative stress. We demonstrated that exogenous glucose mitigated the photo-oxidative damage of cells exposed to 300 μmol photons m^–2^ s^–1^ photosynthetic active radiation. Photosynthesis carbon assimilation scarcely contributed to the cell growth in the presence of glucose, but the photosynthetic apparatus was nevertheless maintained and protected by glucose in a concentration-dependent manner. Supplementation of glucose increased expression of the L-gulonolactone oxidase gene essential for ascorbic acid biosynthesis, whereas no enhanced expression of the genes involved in carotenoid or tocopherol biosynthesis was observed. Under the photo-oxidative stress condition, the ascorbic acid content was strongly enhanced by exogenous glucose. We propose that the biosynthesis of ascorbic acid is one of the major photoprotective processes induced by exogenous glucose. The elucidation of how ascorbic acid is involved in scavenging reactive oxygen species provides key insights into the photoprotective mechanism in red algae.

## Introduction

Cyanidiophyceae is a unique group of red algae, most members of which occupy hot sulfur springs and are well-adapted to low pH (pH 0–5), high temperature (up to 63°C), and high concentrations of heavy metals (Doemel and Brock, 1971; Gross et al., 1998; Gross and Oesterhelt, 1999). Of the three genera—*Galdieria*, *Cyanidioschyzon*, and *Cyanidium*—constituting Cyanidiophyceae, *Galdieria* is facultative heterotrophic and can utilize more than 50 organic carbon sources for growth (Rigano et al., 1977; Gross and Schnarrenberger, 1995). Capable of growing in acid thermal environments and of utilizing various organic carbons, *Galdieria* exhibits great potential for use in biotechnological applications, particularly as a microorganism for nutrient removal in wastewater treatment, for phycocyanin production on food waste hydrolyzates, and for the recovery of rare earth metal elements (Minoda et al., 2015; Henkanatte-Gedera et al., 2017; Sloth et al., 2017).

Since *Galdieria* spp. are facultative heterotrophs, the regulation and transition of nutritional states between heterotrophy and photoautotrophy under various conditions have been widely discussed. Heterotrophic growth has been proposed as an essential survival strategy inside rocks where photosynthesis cannot take place (Gross, 1999; Gross and Oesterhelt, 1999). Despite the dissolved organic carbon (DOC) content in rocks inhabited by *Galdieria* being generally low, uptake of DOC for growth is supposedly achieved using nearby dead cell materials that can be readily hydrolyzed (Gross and Oesterhelt, 1999; Oesterhelt et al., 2007). In laboratory cultivation, even when both light and exogenous glucose were made available, heterotrophic growth rather than mixotrophic growth remained the predominant growth mode (Oesterhelt et al., 2007). Whether in a condition of illumination or darkness, externally supplied sugar repressed photosynthetic capacity by decreasing the amount of PSII and several Calvin–Benson–Bassham (CBB) cycle enzymes, including ribulose-1,5-bisphosphate carboxylase/oxygenase (Rubisco; Oesterhelt et al., 2007; Tischendorf et al., 2007). Although the cellular content of the photosynthetic apparatus was largely reduced, photosynthetic pigments, such as chlorophyll (Chl) *a* and carotenoids, were still present in the light (Gross and Schnarrenberger, 1995; Stadnichuk et al., 1998; Oesterhelt et al., 2007). Once these pigments absorb excessive light, excitation energy influx outpaces the downstream photosynthetic processes, leading to the accumulation of reactive oxygen species (ROS) and subsequently photo-oxidative damage (Apel and Hirt, 2004). How these facultative heterotrophic cyanidiophytes react to strong light and their underlying acclimation strategies have not yet been thoroughly investigated.

Numerous photoprotective mechanisms involved in the regulation of the ROS level are induced by strong irradiation (Sirikhachornkit and Niyogi, 2010). Several mechanisms are employed to dissipate excess excitation energy and thus prevent ROS generation. On the other hand, accumulated ROS can be detoxified through exposure to antioxidants, such as carotenoids, tocopherols, and ascorbic acid are known to function as ROS scavenger.

The molecular aspect of photoprotective mechanisms has been extensively identified under photoautotrophic growth conditions, but the processes induced by exogenous organic carbon to counteract photo-oxidative damage are less characterized. The photoprotective mechanisms of *Galdieria* under mixotrophic cultivation are of particular interest because many *Galdieria* isolates have been reported to be sensitive to light and incapable of growing under photoautotrophic conditions at >200 μmol photons m^–2^ s^–1^ photosynthetic active radiation (PAR) (Sloth et al., 2006; Oesterhelt et al., 2007; Thangaraj et al., 2011). Moreover, environmental DNA surveys have revealed that most members of *Galdieria* primarily occupy low-light conditions such as sulfur fume and endolithic and interlithic habitats (Ciniglia et al., 2004; Skorupa et al., 2013; Hsieh et al., 2015, 2018). Recent environmental surveys revealed that, contrary to what is usually expected, sunlight-exposed soils sometimes contain abundant populations of *Galdieria partita* (Hsieh et al., 2018). A high DOC of these soils (Supplementary Table S1) raises the possibility that exogenous organic carbon sources protect cells from photo-oxidative damage. In support of this hypothesis, examining the effect of exogenous glucose on cell growth can facilitate the identification of the key component conferring protection against photo-oxidative damage and may advance our understanding of photoprotection activated by exogenous organic carbon. In addition, the results are expected to help optimize microalgal growth conditions for biotechnological applications such as biofuels in general.

To identify the glucose-induced photoprotective process, we obtained an isolated *G. partita* THAL043 strain collected from the Tatun Volcano Group of Taiwan and examined the effects of glucose under various light conditions. A strong light condition was established in which cells were photodamaged without exogenous organic carbon but partially recovered upon supplementation of the cultivation with glucose. Pigment quantification, whole-transcriptome shotgun sequencing (RNA-Seq), and RT-qPCR were employed to identify the antioxidants induced by glucose. Analyses of fluorescence and P700 were conducted to identify the ROS accounting for photoinhibition. Our results suggest that biosynthesis of ascorbic acid is induced by glucose to protect against photoinhibition and photo-oxidative damage under strong illumination.

## Materials and Methods

### Growth *Conditions* of *Galdieria partita*

Axenic cultures of *G. partita* THAL043 were isolated from a sample collected at a small stream (pH: 2.15; Temperature: 46–48°C) at DaYouKeng in the Tatun Volcano Group area in Taiwan (Hsieh et al., 2015). The *rbc*L sequence is a more widely used DNA barcode for the species identification in Cyanidiophyceae (e.g., Hsieh et al., 2015). The species identification of our materials was therefore verified based on the comparison of its *rbc*L sequence against the GenBank database of the National Center for Biotechnology Information (NCBI). The newly generated *rbc*L sequence was deposited to the GenBank database (accession number: MK239148). The isolated strain was deposited and maintained in the Tung-Hai Algal Lab (THAL) Culture Collection at the Tunghai University, Taichung, Taiwan (available upon request^1^). Cells were grown in modified Allen’s (MA) medium (pH adjusted to 2.0 with H_2_SO_4_) (Minoda et al., 2004) at 40°C with shaking at 125 rpm under continuous cool-white fluorescent light. Light intensity was set to 300 μmol photons m^–2^ s^–1^ for HL and 20 μmol photons m^–2^ s^–1^ for LL. To simulate the dark condition, Erlenmeyer flasks were wrapped with aluminum foil. In the time-course experiments, cells acclimated to the LL condition were harvested and resuspended at an initial algal concentration equivalent to 10^7^ cells mL^–1^. For survival tests on solid medium, cells were resuspended in water and deposited as drops (2 × 10^4^ cells) on MA agar plates (1.2% w/v). Cells were then grown under the LL condition for 14 days.

### Extraction and Quantification of Chl *a* and Carotenoids

To estimate the Chl *a* content, 1.5 mL of the harvested cells was resuspended using an equal amount of DMSO and incubated at 65°C for 10 min. After centrifugation at 13,000 × *g* for 1 min, the supernatant was measured using a Hitachi U-3900 UV-visible spectrophotometer. The Chl *a* concentration was estimated using the equation Chl *a* (μg/mL) = (*A*_665.1 nm_ – *A*_750 nm_) ÷ 0.0834, based on a calibration curve of authentic standard (Cat. No. C5753, Sigma-Aldrich) (Supplementary Figure S1). The Chl *a* content was calculated as the Chl *a* concentration multiplied by the cell density as assessed using a hemocytometer.

The profiles and cellular content of carotenoids were assessed through HPLC. Pigments were extracted according to the method of Cunningham et al. (2007) with modifications. In brief, cells resuspended in 300 μL of acetone:methanol 7:2 (v/v) with 0.1 g of sea sand were vortexed for 15 min. After centrifugation, 200 μL of the extract was transferred to another tube, and the pellet was extracted using 300 μL of ethyl acetate for 15 min. This second extract (300 μL) was combined with the first and 400 μL of deionized water was added; the solution was gently mixed. After completing phase separation through centrifugation, the upper phase was transferred to a new microfuge tube for HPLC analysis on a Hitachi Chromaster system with a Chromaster 5430 diode array detector (Hitachi).

The extracted pigments were eluted on a 150 mm × 2.1 mm PrincetonSPHER-C30 column (Princeton Chromatography, Inc.) at a flow rate of 0.6 mL min^–1^. A gradient of 0 to 30% mobile phase B (methanol:methyl tert-butyl ether:water = 7:90:3) in mobile phase A (methanol:methyl tert-butyl ether:water = 81:15:4) over 15 min was used for pigment separation. Chl *a*, zeaxanthin, β-cryptoxanthin, and all-*trans*-β-carotene were identified using authentic standards (zeaxanthin [Cat. No. ASB-00026504-005] was obtained from ChromaDex, Inc.; β-cryptoxanthin [Cat. No. C6368] and all-*trans-*β-carotene [Cat. No. 9750] were purchased from Sigma-Aldrich). A clear peak that was eluted immediately after all-*trans*-β-carotene was identified as 9-*cis*-β-carotene (Cunningham et al., 2007; Gupta et al., 2015). Concentrations of the identified pigments were estimated based on the peak area detected at 436 nm and calculated using the calibration curves obtained from five different concentrations of the standards (Supplementary Figure S2). The amount of 9-*cis*-β-carotene was quantified using the calibration curves of all-*trans*-β-carotene because the extinction coefficients of 9-*cis*- and all-*trans*-β-carotene are congruous (Schierle et al., 2004). The amount of total carotenoids was calculated as the sum amount of zeaxanthin, β-cryptoxanthin and β-carotene (all-*trans* and 9-*cis* forms). The carotenoid content was calculated as the molar ratio of carotenoids to Chl *a* multiplied by the Chl *a* content.

### Transmission Electron Microscopy (TEM)

Algal cells grown after 6 days in the time-course experiments were harvested and fixed using a high pressure freezing system (EM PACT2, Leica) at 2000–2050 bar. Freeze substitution in anhydrous acetone containing 1% OsO_4_ and 0.1% uranyl acetate was conducted using a Leica EM AFS2 system. Samples were maintained at −85°C for 3 days, followed by −60, −20, and 0°C for 1 day each; finally, the samples were kept at room temperature. After rinsing with acetone for 12 h twice, the samples were infiltrated and embedded in Spurr’s resin or LR gold resin. Ultrathin sections were stained with uranyl acetate (5%) in 50% methanol for 10 min and then stained with lead citrate (0.5%) for 4 min. Sections were examined using a Tecnai G2 Spirit TWIN microscope (FEI Company).

### Determining δ^13^C and Estimating the Photosynthetic Fraction of Carbon Biomass

Cells grown in MA medium for 6 days were harvested and dried at 55°C for 48 h, and an aliquote (2 mg) was submitted for stable carbon isotope analysis using a FlashEA 1112 nitrogen and carbon analyzer connected to a DELTA V Advantage isotope ratio mass spectrometer (Thermo Fisher Scientific). The δ^13^C values were calculated as [(R_sample_ ÷ R_standard_) – 1] × 1000 (‰), where R_sample_ is the ^13^C:^12^C ratio of the sample, and R_standard_ = 0.0112372 (Hayes, 1983). The photosynthetic fraction of carbon biomass was calculated using the equation presented by Heifetz et al. (2000): (δ^13^C_hetero_ – δ^13^C_sample_)/(δ^13^C_hetero_ – δ^13^C_auto_), where δ^13^C_sample_ is the δ^13^C value of the sample cells, and δ^13^C_hetero_ and δ^13^C_auto_ are the average δ^13^C values of the cells grown in the dark + glu and LL conditions, respectively.

### RNA-seq and RT-qPCR Analyses

Total RNA was isolated by solublizing cells in 1% (w/v) *N*-lauroylsarcosine in the TRI Reagent (Sigma-Aldrich) and then using a Direct-zol RNA MiniPrep kit (Zymo Research). For RNA-seq, paired-end stranded mRNA samples prepared under five growth conditions (dark, LL, dark + glu, LL + glu, HL + glu) for 6 days were submitted to an Illumina HiSeq 2500 Sequencer. The raw output sequence data (3.7–4.2 Gbases per sample) were deposited into the Sequence Read Archive of NCBI (SRA accession: PRJNA507473), and the basic metrics of the data is shown in Supplementary Table S3. *De novo* transcriptome assembly was conducted on Trinity v. 2.6.6 (Grabherr et al., 2011). Gene coding regions in the assembly were identified using TransDecoder (Haas et al., 2013), and their functions were annotated using GhostKOALA (Kanehisa et al., 2016). Differential expression analysis was performed using RSEM (Li and Dewey, 2011) to identify housekeeping genes constitutively expressed with low variance (LogFC values between −0.2 and 0.2 in all the five samples). The coding region sequences and the corresponding primer sequences used for RT-qPCR are listed in Supplementary Table S4.

For RT-qPCR analysis, cDNA was synthesized from total RNA using a SuperScript IV First-Strand Synthesis System (Invitrogen). Quantitative PCR based on SYBR-Green fluorescence was performed, and relative quantification of cDNA was analyzed using the Thermo Fisher Cloud service.

### Quantification of Ascorbic Acid

Ascorbic acid was determined using a calorimetric method described by Shigeoka et al. (1987) with modifications. Harvested cells suspended in 5% (w/v) metaphosphoric acid were frozen once and disrupted with a Bioruptor Pico sonicator (Diagenode) for 6 × 20 s with each interval of 30 s. After centrifugation at 15,000 × *g* for 10 min at 4°C, the homogenates (500 μL) were mixed with 100 μL of 6 mM 2,6-dichlorophenolindophenol (DCPIP). After incubation at room temperature for 20 min, 250 μL of 2% (w/v) thiourea in 2% (w/v) metaphosphoric acid was added. The mixture was incubated at 50°C for 1 h with 125 μL of 2% (w/v) 2,4-dinitrophenylhydrazine in 25% (v/v) sulfuric acid. After 625 μL of 85% (v/v) sulfuric acid was added to the mixture on ice, the resulting sample was determined at 520 nm.

The cellular content of ascorbic acid was also estimated using an ascrobate oxidase based-method (ab219928 Ascorbic Acid Assay Kit, Abcam). Harvested cells were suspended in 1 mL of the phosphate-buffered saline (PBS) solution (137 mM NaCl, 2.7 mM KCl, 8 mM Na_2_HPO_4_, and 2 mM KH_2_PO_4_, pH 7.4) and centrifuged at 18,000 × *g* for 1 min at 4°C twice to remove residual ascorbic acid in the growth medium. The resulting cell pellets resuspended in 100 μL of PBS solution with 0.05 g of sea sand were frozen using liquid nitrogen. Cells were disrupted by vortexing for 10 min at 4°C. After centrifugation, 50 μL of the extract was transferred to the assay reaction mix, and the fluorescence intensity was measured, as per manufacturer’s instructions.

### Analyses of Fluorescence and P700

Cells grown in the MA medium were harvested and placed in the dark for at least 30 min. Fluroescence and P700 were separately measured using a Dual-PAM-100 system (Heinz Walz) equipped with a stirred cuvette at 40°C. Cells were adjusted with MA medium to 10 μg Chl/mL for fluorescence analysis and to 20 μg Chl/mL for P700 analysis. The maximum photochemical efficiency of PSII was estimated from F_V_/F_M_, which is equivalent to (F_M_ - F_0_) ÷ F_M_, where F_M_ is the maximum fluorescence level measured while applying a 600-ms saturation pulse (12,000 μmol photons m^–2^ s^–1^) and F_0_ is the minimum fluorescence level. P700 was measured using a dual wavelength unit (830/875 nm), and the photo-oxidizable P700 was obtained by applying a 250-ms saturation pulse under far-red illumination (Klughammer and Schreiber, 1994, 2008). To eliminate unstable P700 readings caused by stirring, the stirrer was turned off 5 s before the P700 measuring procedure was applied.

### Statistical Analysis

Experimental data are expressed as mean values ± SD of three independent experiments. One-way analysis of variance (ANOVA) followed by Tukey’s multiple comparison test at an α = 0.05 significance level were performed to determine significant differences between any two growth conditions or two treatments at the same time point using Origin 2017 (OriginLab).

## Results

### Glucose-Induced Cell Survival and Growth Recovery Under the High Light Condition

To determine light sensitivity of the isolated *G. partita* strain, cells were grown under three continuous light conditions: darkness, low light (LL), and high light (HL; equivalent to 0, 20, and 300 μmol photons m^–2^ s^–1^ PAR, respectively). Heterotrophic (dark) and mixotrophic (LL and HL) cultivations were performed with 25 mM of supplemented glucose (hereafter referred to as + glu unless a different glucose concentration is indicated).

**FIGURE 1:**
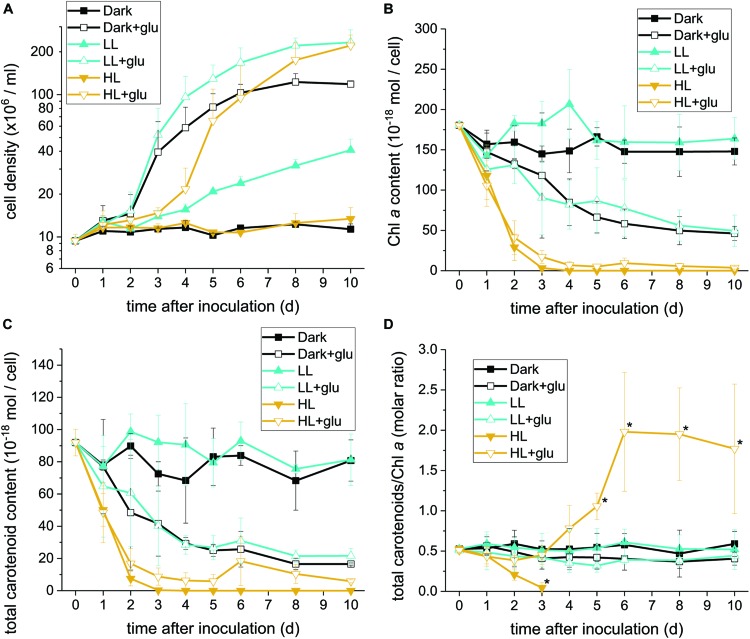
Proliferation curves with changes in the Chl *a* and carotenoid contents. **(A)** Cell proliferation curves in the dark, LL, and HL conditions (equivalent to 0, 20, and 300 μmol photons m^–2^ s^–1^ PAR, respectively). The growth medium supplemented with 25 mM of glucose is denoted by ‘+glu.’ Cellular content of Chl *a* **(B)** and total carotenoids **(C)** as well as the molar ratio of carotenoids to Chl *a* **(D)** were assessed. Cells were inoculated to the cell density equivalent to 10^7^ cells mL^–1^ and grown at 40°C with shaking at 125 rpm. Data are expressed as mean ± SD of three independent experiments. Asterisks indicate a significance difference from the other growth conditions of the same day as determined using one-way ANOVA followed by Tukey’s multiple comparison test.

**FIGURE 2:**
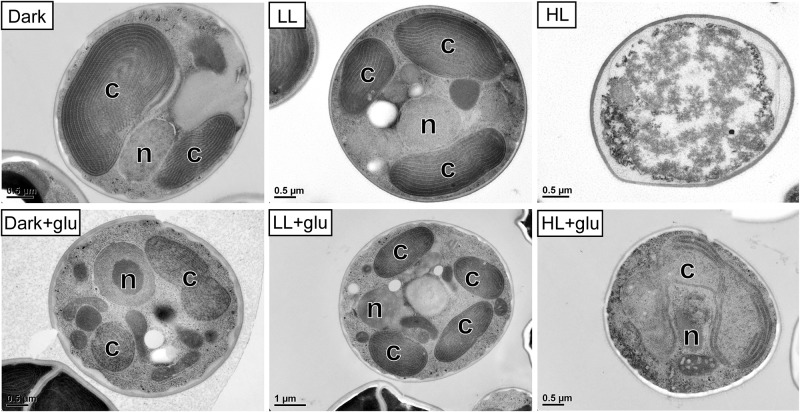
Transmission electron micrographs of cells grown for 6 days. The growth conditions are the same as described in Figure 1. n, nucleus; c, chloroplast. No organelles were observed under the HL condition without glucose supplementation.

Without externally supplied glucose, *G. partita* proliferated only in the LL condition (Figure 1A). Organelles disappeared in almost all cells acclimated to HL within 6 days, whereas both dark- and LL-acclimated cells retained complete thylakoid membrane networks (Figure 2). These micrographs provide concrete evidence that cells exposed to HL undergo photodamage and subsequently perish.

When the cultivations were supplemented with glucose, cells proliferated much faster than under the phototrophic conditions (Figure 1A). Most strikingly, HL-exposed *G. partita* resumed propagating and its cellular structure partially, exhibiting a few layers of thylakoid membrane in the chloroplasts (Figure 2). The overall results suggest that *G. partita* under the high light stress is protected by supplementation of glucose.

### Retention of Chl *a* and Carotenoids Under the HL or Glucose-Supplemented Condition

As the chloroplast ultrastructure drastically changed under the HL condition with or without glucose supplementation, we further analyzed the Chl *a* content to obtain a simple estimate of the number of photosystems. The carotenoid content, which act both as accessory photosynthetic pigments and photoprotectants of photosynthetic apparatus, was also determined. The HPLC chromatogram yielded a carotenoid profile that identifies zeaxanthin and β-carotene as the two major species (Supplementary Figure S3). Antheraxanthin or violaxanthin is not present in the chromatogram, and the gene encoded for zeaxanthin epoxidase, essential to producing antheraxanthin and violaxanthin, was not found in the transcriptome sequence database generated and deposited in NCBI (SRA accession: PRJNA507473). The zeaxanthin epoxidase is commonly absent in most red algae and exceptionally present in several species in Compsopogonophyceae and Florideophyceae (Dautermann and Lohr, 2017). In addition, the carotenoid profile is the same as that of other members of Cyanidiophyceae (Takaichi et al., 2016).

Under the HL condition, Chl *a* decayed to non-detectable levels within 4 days, indicating that the photosynthetic apparatus was readily degraded (Figure 1B). Although the presence of glucose did not prevent the photosynthetic apparatus from rapidly decaying, the Chl *a* content remained at a low level, indicating that the photosynthetic apparatus was still present. By contrast, when cells acclimatized to the dark + glu or LL + glu condition, the Chl *a* content declined to approximately one-third of their initial levels, as likely reflected by the smaller chloroplast size and highly proliferated cells visible through electron microscopy (Supplementary Figures S4, S5).

The decay pattern of carotenoids, either respectively or collectively determined, was identical to that of Chl *a* (Figure 1C and Supplementary Table S2). Nevertheless, using the molar ratio of carotenoids to Chl *a* as an estimate, the rate of carotenoid decay differed significantly from that of Chl *a* decay under the HL condition (Figure 1D). In the absence of glucose, carotenoids decayed more rapidly than did Chl *a*. By contrast, the ratio remained steady over the first 3 days under the HL + glu condition and then increased thereafter. The ratio of β-carotene or zeaxanthin to Chl *a* exhibited a trend similar to that of total carotenoids, although β-carotene changed more substantially than did zeaxanthin, as evidenced by the molar ratio of β-carotene to zeaxanthin (Supplementary Figure S6). The overall results suggest that regardless of the light condition, the cellular content of the photosynthetic apparatus decreases because of exogenous glucose. Changes in the proportion of carotenoids relative to Chl *a* under HL likely represent a consequence of the interplay between light-induced photodamage and glucose-induced photoprotection.

### Heterotrophic Growth in Both the LL + glu and HL + glu Conditions

The retention of the photosynthetic apparatus in the HL + glu condition raises a question concerning the nutritional state in mixotrophic cultivations. Although the nutritional state may be a state between photoautotrophy and heterotrophy, *Galdieria* was considered exclusively heterotrophic when sufficient glucose was supplied in medium light conditions (Oesterhelt et al., 2007). Given that Rubisco prefers ^12^CO_2_ as its substrate (O’Leary, 1988), we used the δ^13^C value to assess the ratio between heterotrophic and photoautotrophic contributions to carbon biomass. Cells under the photoautotrophic LL condition had a high negative δ^13^C level, and incubation in the heterotrophic dark + glu condition for 6 days shifted δ^13^C to a low negative level close to that of glucose (Table 1). Provided that the photosynthetic fraction of carbon biomass—calculated based on δ^13^C—in the photoautotrophic and heterotrophic conditions was set to 100 and 0%, respectively, the carbon biomass was almost completely attributed to heterotrophic carbon assimilation under both LL + glu and HL + glu conditions. The extent of photosynthetic fraction of carbon biomass equivalent to 6∼7% in the HL + glu condition may have been resulted from the biomass increment before glucose was apparently taken into the cell after 3 days (Table 2). These results support that heterotrophy is the predominant nutritional state in mixotrophic cultivations even under strong light.

**TABLE 1:** δ^13^C and photosynthetic fraction of carbon biomass from cells grown for 6 days.

	**δ^13^C (‰)**	**Photosynthetic fraction of carbon biomass (%)**
Glucose (originated from *Zea mays*)	−10.37 ± 0.00^a^	–
LL (photoautotrophic)	−24.31 ± 0.27^b^	100.00 ± 1.97^b^
Dark + glu (heterotrophic)	−10.68 ± 0.04^a^	0.00 ± 0.27^a^
LL + glu	−10.78 ± 0.02^a^	0.72 ± 0.18^a^
HL + glu	−11.62 ± 0.22^c^	6.89 ± 1.62^c^

*The photosynthetic fraction of carbon biomass is estimated using the average δ^13^C values in the dark + glu and LL conditions set to 0 and 100%, respectively. Data are expressed as average ± SD of three independent experiments. Superscript letters indicate significantly different groups as determined using one-way ANOVA followed by Tukey’s multiple comparison test.*

**TABLE 2:** Changes in glucose concentration in the growth medium under the HL condition.

	**Time after inoculation (d)**		
	
	**0**	**3**	**6**
	
**Initial glugose supplemented (mM)**	**Glucose concentration in the medium (mM)**		
0.25	0.26 ± 0.00	n.d.	n.d.
2.5	2.44 ± 0.10	1.35 ± 0.51	0.29 ± 0.35
25	25.51 ± 0.09	26.40 ± 0.24	17.99 ± 1.03
100	95.68 ± 1.32	98.25 ± 0.29	91.52 ± 5.57

*Data are expressed as average ± SD of three independent experiments. n.d., not detectable.*

### Glucose-Induced Photoprotection Compromised by Norflurazon

**FIGURE 3:**
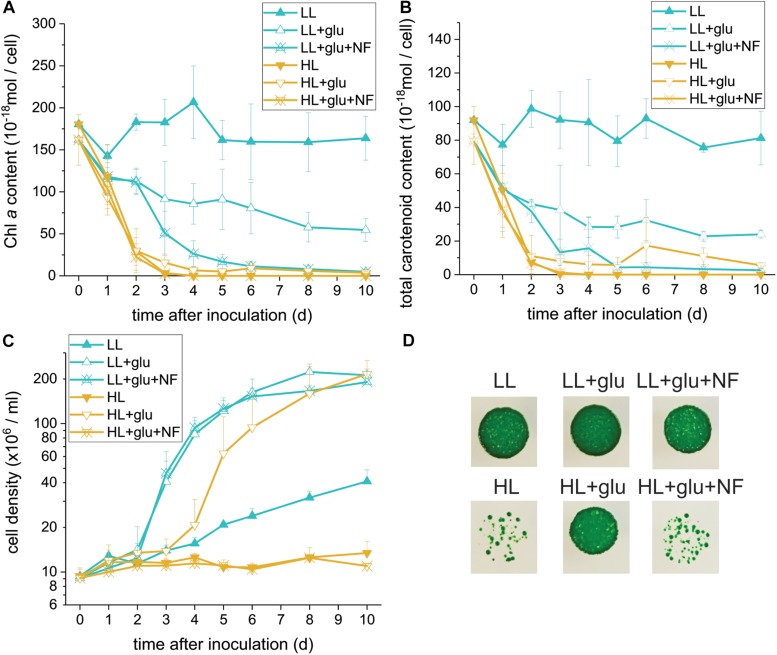
Compromising effects of the carotenoid biosynthesis inhibitor norflurazon (NF) on *G. partita* grown under the HL + glu condition. **(A)** Cell proliferation curves in LL and HL conditions. In the mixotrophic cultivation supplemented with 25 mM of glucose (+ glu), ‘+NF’ denotes 5 μM of norflurazon supplemented in the growth medium. Cellular content of Chl *a* **(B)** and total carotenoids **(C)** were assessed. Data are expressed as average ± SD of three independent experiments. **(D)** Survival test of cells (2 × 10^4^ cells) in the indicated growth conditions for 6 days. Pictures were captured 14 days after cells were deposited on MA agar plates.

Because photosynthesis contributed little to cell mass production under the HL + glu condition, we used norflurazon treatment to further assess whether the photosynthetic apparatus and its accompanying photoprotection was essential. Norflurazon is a phytoene desaturase inhibitor that depletes carotenoids and leads to the degradation of PSII in *Galdieria* (Marquardt, 1998). A low concentration of norflurazon (5 μM) was supplied (referred to as + NF) such that photosynthetic apparatus, including carotenoids, was completely depleted under the HL + glu condition and mildly suppressed under the LL + glu condition (Figures 3A,B). Cell proliferation was scarcely affected by norflurazon under LL (Figure 3C), which was anticipated because photosynthesis does not contribute to cell growth when heterotrophic growth is predominant. By contrast, under HL, norflurazon compromised glucose-induced recoveries of cell proliferation and survival (Figures 3C,D); consistent with this result, the photosynthetic apparatus was maintained and protected in response to strong light even though autotrophic carbon assimilation contributed little to cell mass production under the HL + glu condition.

### Glucose Concentration-Dependent Photoprotection Under the HL Condition

**FIGURE 4:**
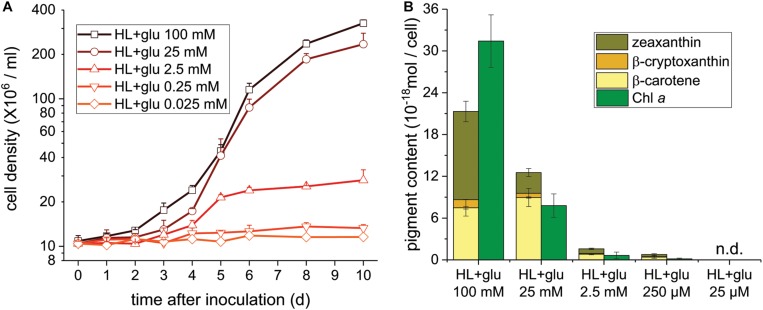
Cell proliferation and contents of Chl *a* and carotenoids supplemented of the indicated glucose concentrations under the HL condition. **(A)** Cell proliferation curves. **(B)** Cellular content of Chl *a* and total carotenoids 10 days after inoculation. Data are expressed as mean ± SD of three independent experiments. n.d., not detectable.

To further assess the threshold concentration of glucose that could induce photoprotection, a wide range of glucose concentrations (0.025 to 100 mM) were applied to the HL cultivation. The minimum glucose concentration for cell proliferation under the HL condition was approximately 0.25 mM, since no significant cell proliferation was observed after 0.25 mM of glucose was supplied as well as after the initial 2.5 mM of glucose was reduced to approximately 290 μM after 6 days (Figure 4A and Table 2). Even when 0.25 mM of glucose was readily consumed within 3 days, the cells survived until the end of the experiment, as indicated by the presence of photosynthetic pigments (Figure 4B). These results demonstrate that an initial glucose concentration as low as 2.5 mM enables cells to effectively resist photo-oxidative damage for at least 10 days.

The photoprotective effect of exogenous glucose was also demonstrated by the increase in the Chl *a* and carotenoid contents with an increase in the glucose concentration. When a sufficient amount of glucose (25 or 100 mM) was supplied, the Chl *a* content drastically increased at 100 mM of glucose, but the cell proliferation rate did not significantly increase (Figures 4A,B). Furthermore, zeaxanthin became the dominant carotenoid species when the cultivation was supplemented with 100 mM of glucose, whereas the zeaxanthin concentration was lower than the β-carotene concentration in the presence of lower glucose concentrations (25 and 2.5 mM; Figure 4B).

### Ascorbic Acid Biosynthesis Induced by Glucose

**FIGURE 5:**
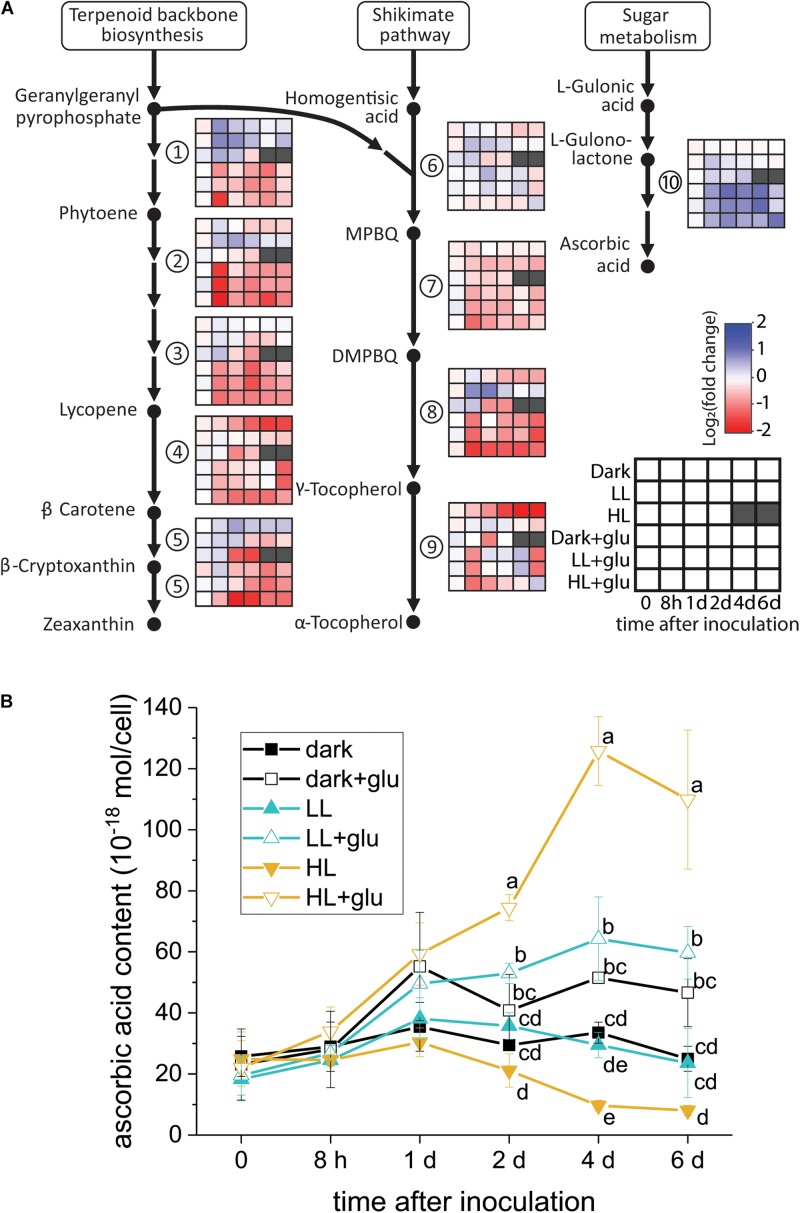
Analysis of antioxidant biosynthesis activated by exogenous glucose. **(A)** RT-qPCR analysis of transcripts encoded for biosynthesis of carotenoids, tocopherols, and ascorbic acid. The fold change is calculated as the expression level of transcripts compared with the average level of all six conditions upon inoculation. The housekeeping genes used for reference expression are DNA topoisomerase I (EC 5.99.1.2) and ubiquitin carboxyl-terminal hydrolase 25 (EC 3.4.19.12). Data are expressed as mean of three independent experiments. (1) 15-*cis*-phytoene synthase (EC 2.5.1.32); (2) 15-*cis*-phytoene desaturase (EC 1.3.5.5); (3) zeta-carotene desaturase (EC 1.3.5.6); (4) lycopene beta-cyclase (EC 5.5.1.19); (5) beta-carotene hydroxylase (EC 1.14.13.-); (6) homogenitisate phytyltransferase (EC 2.5.1.115); (7) tocopherol cyclase (EC 5.5.1.24); (8) MPBQ/MSBQ methyltransferase (EC 2.1.1.295); (9) tocopherol *O*-methyltransferase (EC 2.1.1.95); (10) L-gulonolactone oxidase (EC 1.1.3.8). **(B)** Changes in the cellular content of ascorbic acid using a calorimetric method (Shigeoka et al., 1987; see section “Materials and Methods” for details). The growth medium supplemented with 25 mM of glucose is denoted by ‘+glu.’ Data are expressed as average ± SD of three independent experiments. Lowercase letters indicate significantly different groups of the same time as determined using one-way ANOVA followed by Tukey’s multiple comparison test.

Although several lines of evidence support that photoprotection is induced by glucose, the putative photoprotective process linked to glucose metabolism remains unknown. One possible process is the accumulation—resulting from highly expressed genes encoding enzymes involved in antioxidant biosynthesis—of certain antioxidants known to have photoprotective effects. To test whether this process occurred, the temporal expression of the genes involved in the biosynthesis of several antioxidants consisting of carotenoids, tocopherols, and ascorbic acid was analyzed. As genomic sequences of *G. partita* are not yet available, RNA-Seq and *de novo* transcriptome assembly were employed to annotate genes prior to RT-qPCR analysis (see section “Materials and Methods” for details). Among the examined genes, only the L-gulonolactone oxidase gene involved in ascorbic acid biosynthesis was highly expressed upon glucose supplementation, whereas the expression of the genes involved in biosynthesis of carotenoids and tocopherols were mostly suppressed by glucose (Figure 5A). Transcriptome analysis also revealed that the L-gulonolactone oxidase gene was upregulated when glucose was supplemented for 6 days (Supplementary Table S5). Expression of the L-galactose dehydrogenase was not apparently upregulated by glucose supplementation; many ascorbate-related oxygen scavenging genes, such as superoxide dismutase and ascorbate peroxidase, were highly expressed in the LL and HL + glu conditions (Supplementary Table S5). Downregulation of genes for carotenoid biosynthesis is consistent with the low cellular content of carotenoids in the presence of glucose (Figure 1C). The cellular content of ascorbic acid was indeed increased by glucose, achieving the highest accumulation of ascorbic acid detected under the HL + glu condition (Figure 5B). Furthermore, the glucose concentration-dependent increase in the ascorbic acid level under the HL condition was confirmed using an ascorbate-specific enzyme-based assay (Supplementary Figure S7), suggesting the involvement of ascorbic acid in the glucose concentration-dependent photoprotection. These results suggest that ascorbic acid biosynthesis is regulated both transcriptionally by glucose and post-transcriptionally in response to light and glucose.

### Photoprotective Response Elicited by the ${\text{O}}_{2}^{●-}$ Scavenger and Ascorbic Acid

**FIGURE 6:**
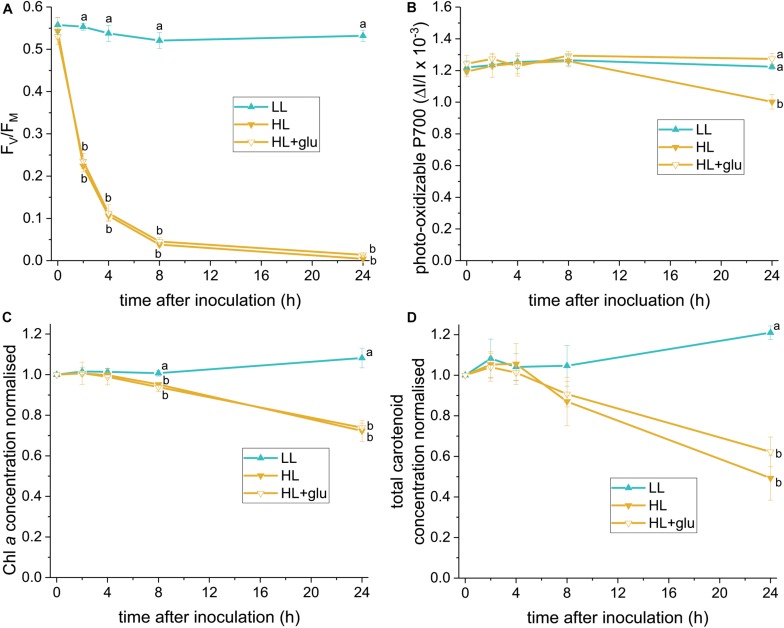
Photoprotective response exerted by supplemented glucose over 24 h of the HL condition. Several photosynthetic parameters were measured to determine an early indicator of the photoprotective response exerted by the supplemented glucose (25 mM) in the HL condition. **(A)** F_V_/F_M_. **(B)** Photo-oxidizable P700 level at the same Chl *a* concentration. **(C,D)** Are the concentrations of Chl *a* and total carotenoids, respectively, calculated as mol/mL of the growth medium and normalized to the initial level upon inoculation. Values measured under the LL condition are used as reference without photoinhibition. Data are expressed as average ± SD of three independent experiments. Lowercase letters indicate significantly different groups of the same time as determined using one-way ANOVA followed by Tukey’s multiple comparison test.

The glucose-induced biosynthesis of ascorbic acid prompted us to evaluate whether ascorbic acid is an effective protectant against the HL condition. To identify a reliable indicator of photoprotective response, PSII and PSI activities were assessed using F_V_/F_M_ and photo-oxidizable P700 levels, respectively, under LL and HL conditions. Photoinhibition of PSII, demonstrated by a decrease in the F_V_/F_M_ value, started rapidly within 1 h under the HL condition (Figure 6A). Photoinhibition of PSI, as indicated by a lower photo-oxidizable P700 level at the same Chl *a* concentration, started after 24 h (Figure 6B). Decay of the Chl *a* and carotenoid contents commenced within 8–24 h (Figures 6C,D).

The earliest photoprotective response induced by glucose under HL was indicated by an unchanged PSI activity level after 24 h. Glucose did not hitherto significantly increase the F_V_/F_M_, Chl *a*, and carotenoid levels. Further recovery of the PSII activity, as indicated by a slight increase of F_V_/F_M_, was detected after 42 h (Supplementary Figure S8A). Moreover, glucose prevented the salient sluggish redox kinetics of P700 (Supplementary Figure S9). After 6 days in the HL + glu condition, F_V_/F_M_ recovered to nearly half the initial level. The photo-oxidizable P700 increased by 50% of the initial level (Supplementary Figure S8B), which might be attributable to a high PSI to PSII stoichiometric ratio in the presence of glucose (Oesterhelt et al., 2007).

**FIGURE 7:**
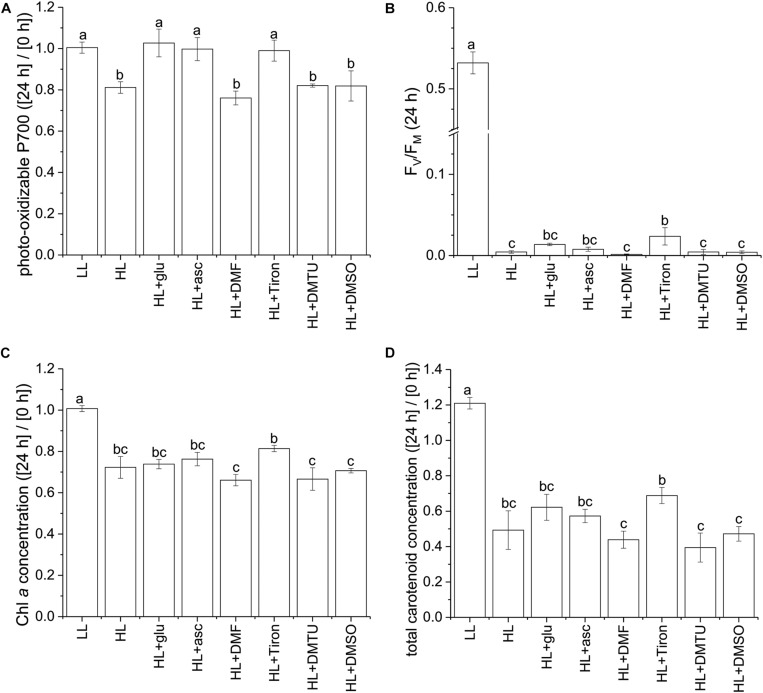
Effects of ascorbic acid, norflurazon, and various ROS-specific scavengers on the photoprotective response under the HL condition. Photo-oxidizable P700 level at 24 h normalized to the initial at 0 h **(A)** is indicative of photoprotective response under the HL condition. Values measured under the LL condition are used as reference without photoinhibition. Ascorbic acid (50 mM) was supplemented to evaluate its role in photoprotection. Norflurazon (5 μM) was applied in the HL + glu condition to examine the compromising effect of carotenoid biosynthesis on glucose-induced photoprotection. DMF (10 mM), Tiron (50 mM), DMTU (50 mM), and DMSO (0.28M) are used as ROS-specific scavengers to eliminate ^1^O_2_, ${\text{O}}_{2}^{●-}$, H_2_O_2_, and OH^⋅^, respectively. **(B)** F_V_/F_M_ at 24 h after inoculation. **(C,D)** Are respectively Chl *a* and total carotenoid concentrations calculated as mol/mL of growth medium and normalized to the initial level upon inoculation. Data are expressed as average ± SD of three independent experiments. Lowercase letters indicate significantly different groups as determined using one-way ANOVA followed by Tukey’s multiple comparison test.

Using the photo-oxidizable P700 level at 24 h as a proxy, we analyzed the photoprotective response induced by exogenous ascorbic acid under the HL condition. Several ROS-specific scavengers were used to infer the corresponding ROS involved in the photoinhibitory process. Supplementation of ascorbic acid led to elevation of the cellular level of ascorbic acid after 24 h (Supplementary Figure S10). In addition, ascorbic acid, as well as the ${\text{O}}_{2}^{●-}$ scavenger sodium 4,5-dihydroxybenzene-1,3-disulfonate (Tiron; Bleeke et al., 2004), exhibited an effective photoprotective response, as indicated by the unchanged P700 level (Figure 7A). By contrast, 2,5-dimethylfuran (DMF), dimethylthiourea (DMTU), and dimethyl sulfoxide (DMSO)—which were used to eliminate ^1^O_2_ (Benderliev et al., 2003), H_2_O_2_ (Foreman and Tarloff, 2008), and OH^⋅^ (Franco et al., 2007), respectively—did little to prevent the photosystems from undergoing photoinhibition. In the HL condition, F_V_/F_M_ was mildly affected by the various supplemented chemicals upon inoculation and was reduced to a low level at 24 h regardless of the chemical treatment (Figure 7B and Supplementary Figure S11). The photo-oxidizable P700 level at 24 h was again a reliable early indicator of the photoprotective response, whereas Chl *a* and total carotenoid content did not significantly recover (Figures 7C,D).

## Discussion

This study characterized the extremophilic red alga *G. partita* under light stress and demonstrated that the photosynthetic apparatus was protected by glucose supplementation even though the cell grew heterotrophically. Among the three groups of antioxidants—ascorbic acid, carotenoids, and tocopherols—commonly accumulating in the chloroplast and analyzed in this study, ascorbic acid is recognized as the crucial antioxidant induced by glucose to protect the photosynthetic apparatus against photoinhibition and photo-oxidative damage. Furthermore, the ${\text{O}}_{2}^{●-}$ scavenge reaction was identified as an effective photoprotective process. Ascorbic acid accumulation is known to enhance ${\text{O}}_{2}^{●-}$ scavenge reactions, either operating non-enzymatically or catalyzed by superoxide dismutase and ascorbate peroxidase (Asada, 2006). The putative involvement of ascorbic acid in the ${\text{O}}_{2}^{●-}$ scavenge process may advance our understanding of the photoprotection mechanism in red algae.

While photosynthetic eukaryotes that utilize both photosynthesis and external organic carbon for growth are widespread (Selosse et al., 2017), *Galdieria* does not seem to perform both nutritional states simultaneously. Regardless of the light condition, glucose induces a change from photoautotrophic growth to heterotrophic growth, as confirmed by the δ^13^C analysis. This switch may involve the depletion of Rubisco (Oesterhelt et al., 2007) and may commence before cell proliferation, as photosynthetic contribution to carbon biomass during growth is barely detectable when glucose is supplied. Although photosynthetic carbon assimilation is heavily suppressed, the photosynthetic apparatus is maintained, but with only a small amount surviving. The reason why photosynthetic apparatus is maintained, however, remains unknown. As the photosynthetic apparatus is still present under the glucose-supplemented conditions, NADPH and ATP may still be produced through light-dependent electron flow and may not be readily consumed by the CBB cycle. If neither NADPH or ATP is recycled, the lack of substrates for NADPH and ATP production inhibit the photosynthetic capacity and promote ROS production, resulting in photoinhibition (Roach and Krieger-Liszkay, 2014). Therefore, photosynthesis must be acclimated to the imbalance between light-dependent reactions and carbon assimilation. The recovery of the photosynthetic apparatus under the HL + glu condition implies that exogenous glucose exerts influence over photoprotection in addition to its apparent function as energy source.

The accumulation of ascorbic acid helps explain the photoacclimation strategy linked to exogenous glucose. The cellular content of ascorbic acid is influenced by light and glucose uptake, both of which can produce hexose precursors for the biosynthesis of ascorbic acid. The necessity of ascorbic acid accumulation in response to photo-oxidative stress is evidenced by the elevated level of ascorbic acid under the HL + glu condition and protection of *G. partita* from photoinhibition by ascorbic acid supplementation. When photosynthesis is heavily inhibited, ascorbic acid is not sufficiently produced through the CO_2_ fixation pathway. The uptake of organic carbon therefore becomes crucial to compensate for the shortage of ascorbic acid. The complementary roles of glucose and photosynthetic assimilates have also been observed in *Arabidopsis* leaves, where the loss of ascorbic acid in the dark could be prevented through glucose supplementation (Conklin et al., 1997).

Two different enzymes that catalyze the terminal reaction for the production ascorbic acid have been identified in eukaryotes. Whereas many animals have L-gulonolactone oxidase, plants and most algae use L-galactonolactone dehydrogenas to synthesize ascorbic acid (Wheeler et al., 1998, 2015; Linster and Van Schaftingen, 2007). *G. partita*, like other *Galdieria* spp. and unlike plants, possesses L-gulonolactone oxidase. It may be expected that the members of *Galdieria* utilized the animal-like biosynthesis pathway to produce ascorbic acid. However, the positional isotopic labeling result suggests that *G. sulphuraria* uses a biosynthesis pathway similar to other rhodophytes (Wheeler et al., 2015). On the other hand, AMR1 and ERF98 were identified as light-dependent regulators on ascorbic acid biosynthesis in *Arabidopsis* (Wang et al., 2013). *G. partita* may utilize different transcriptional regulation mechanisms since genes involved in the ascorbic acid synthesis are regulated by exogenous glucose but less impacted by light intensity. Furthermore, no homologous genes of AMR1 and ERF98 are identified in *G. sulphuraria*. To the best of our knowledge, transcriptional regulation mechanisms of ascorbic acid biosynthesis in rhodophytes remains undiscovered.

Several lines of evidence support that increased biosynthesis of ascorbic acid is an important photoprotective process induced by the combination of glucose and strong light. Regardless of the light conditions applied, the L-gulonolactone oxidase gene essential for biosynthesis of ascorbic acid was upregulated by glucose at the transcriptional level within 8 h. Accumulation of ascorbic acid reaching to a high level within 4 days depends on the light intensity and the concentration of exogenous glucose. The photoprotective effect of ascorbic acid on *G. partita* cells is illustrated by the delayed onset of cell proliferation after 3 days, when the elevated level of ascorbic acid was achieved. In addition, exogenous ascorbic acid prevented photoinhibition of PSI, as probed using the P700 analysis. Since ascorbic acid can directly interact with ^1^O_2_, ${\text{O}}_{2}^{●-}$, and OH^⋅^ to prevent oxidative damage, or act as scavenger of O_2_H_2_ through ascorbate peroxidase (Smirnoff, 2000), the increase in ascorbic acid is likely a crucial protective response to an elevated level of ROS under strong light. The involvement of the enzymatic system to scavenge ${\text{O}}_{2}^{●-}$ to produce H_2_O_2_ via superoxide dismutase and to scavenge H_2_O_2_ via ascorbate peroxidase under HL + glu is supported by the highly expressed superoxide dismutase and ascorbate peroxidase genes. Which specific ascorbate-related oxygen scavenging genes are expressed in chloroplasts deserves worth further study concerning different expression levels of multiple genes encoding the same superoxide dismutase or ascorbate peroxidase in *G. partita*.

In the photosynthetic electron transport chain, ${\text{O}}_{2}^{●-}$ and ^1^O_2_ are the two major ROS involved in photodamage. Formation of ^1^O_2_ originates from the interaction of O_2_ with triplet excited Chl, primarily in PSII, and ${\text{O}}_{2}^{●-}$ is produced by the leakage of electrons to O_2_ at the receptor side of PSI and PSII (Pinnola and Bassi, 2018). In *G. partita*, the electron flux to PSI is heavily suppressed under high illumination, as indicated by fully oxidized P700 persisting during the 250-ms period of a saturation light pulse (see Supplementary Figure S9). Accordingly, the lack of accumulated electrons at PSI produces little amount of ${\text{O}}_{2}^{●-}$. On the other hand, the reduced plastoquinone pool rapidly triggers excitation energy transfer from PSII to PSI (i.e., energy spillover), which is a unique feature of red algae compared with other algae and plants (Kowalczyk et al., 2013; Ueno et al., 2016). Therefore, production of ${\text{O}}_{2}^{●-}$ can be considerably enhanced by the fully reduced acceptor side of PSII. Whether ^1^O_2_ is also an abundant ROS during the photoinhibition in red algae remains arguable. The production of triplet Chl and ^1^O_2_ may be decreased by the lowered excitation energy in PSII due to the energy spillover. Furthermore, the substantial decrease in Chl *a* under the HL condition with glucose supplementation may reduce the potential production of ^1^O_2_. On the other hand, the use of ROS scavengers may cause unexpected deleterious effects and may not scavenge all of the corresponding ROS. Therefore, ^1^O_2_ may still be considered the cause of photoinhibition. Further evaluation of the ^1^O_2_ and ${\text{O}}_{2}^{●-}$ production in the isolated PSII may be necessary to confirm the role of specific ROS in the photoinhibitory process in red algae.

In contrast to ascorbic acid acting as an apparent antioxidant in response to light, carotenoids may play a minor role in photoprotection. In photosynthesis, carotenoids play a dual role in quenching ROS and assembling photosystems (Pinnola and Bassi, 2018). The RT-qPCR analysis result demonstrated that the genes involved in biosynthesis of carotenoids were not upregulated by exogenous glucose under strong light. Furthermore, the distinct lowered carotenoid level upon norflurazon treatment in the presence of exogenous glucose could be observed only after 3 days in the LL condition (see Figure 3), much later than the photoprotection started after 1 day based on the P700 analysis result. Therefore, the increased ratio of carotenoids to Chl *a* in the HL + glu condition is likely the outcome, rather than the cause, of the photoprotective process. The result that norflurazon compromised the recovery of cell growth in the presence of exogenous glucose may have been due to the lack of carotenoids for the *de novo* biosynthesis of photosystems. Other effects of norflurazon, such as the degradation of PSII (Marquardt, 1998) and altered lipid composition in the chloroplast (Abrous-Belbachir et al., 2009), may also lead to the same interpretation that maintenance of the photosynthetic apparatus is crucial in cell survival under strong light.

## Conclusion

Our data suggest that the biosynthesis of ascorbic acid is an acclimation strategy of *G. partita* to mediate photoprotection in the presence of exogenous glucose. To the best of our knowledge, the need of exogenous glucose to transcriptionally activate the biosynthesis of ascorbic acid and thus to enhance accumulation of ascorbic acid in response to light was not clearly revealed before, whereas the positive relationship between the ascorbic acid level and the light intensity has been characterized in plants under photoautotrophic conditions (Smirnoff, 2000). Whether the accumulation of ascorbic acid induced by exogenous organic carbons is a common feature in *Galdieria* surviving under sun light warrants further study. The photoprotective processes involving the scavenging reactions of O_2_^–^ provide a novel mechanistic view of photosynthetic regulation and further the understanding of the molecular mechanism of photoinhibition largely unknown in red alga.

## Data Availability Statement

The datasets generated for this study can be found in the GenBank (accession number: MK239148), NCBI (SRA accession: PRJNA507473).

## Author Contributions

H-YF and Y-RC designed the experiments. H-YF performed the experiments and drafted the manuscript. H-YF, S-LL, and Y-RC analyzed the data and reviewed the manuscript.

## Conflict of Interest

The authors declare that the research was conducted in the absence of any commercial or financial relationships that could be construed as a potential conflict of interest.

## Footnotes

1 http://algae.thu.edu.tw/lab/?page_id=42
