# Supplementary material for: Biosynthesis of Ascorbic Acid as a Glucose-Induced Photoprotective Process in the Extremophilic Red Alga Galdieria partita
Source: Front Microbiol. 2020 Jan 14;10:3005. doi: 10.3389/fmicb.2019.03005 (PMC6971183; doi:10.3389/fmicb.2019.03005)
Supplement: Supplementary file 1 [file Data_Sheet_1.pdf]

Supplementary Material

1 Supplementary Figures and Tables

1.1 Supplementary Figures

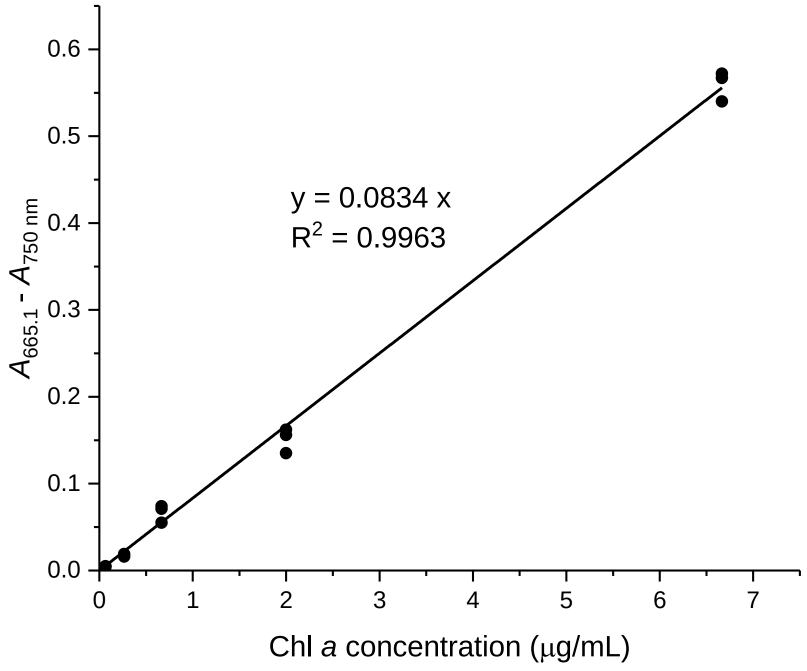

Supplementary Figure S1. Calibration curve of adjusted absorbance ($A_{665.1\ nm} - A_{750\ nm}$) and Chl *a* concentration.

Black dots denote the adjusted absorbance of five concentrations of Chl *a* in three replicates, and the black line indicates the calibration curve obtained through linear regression. The equation of the linear regression curve is used to estimate the Chl *a* concentration.

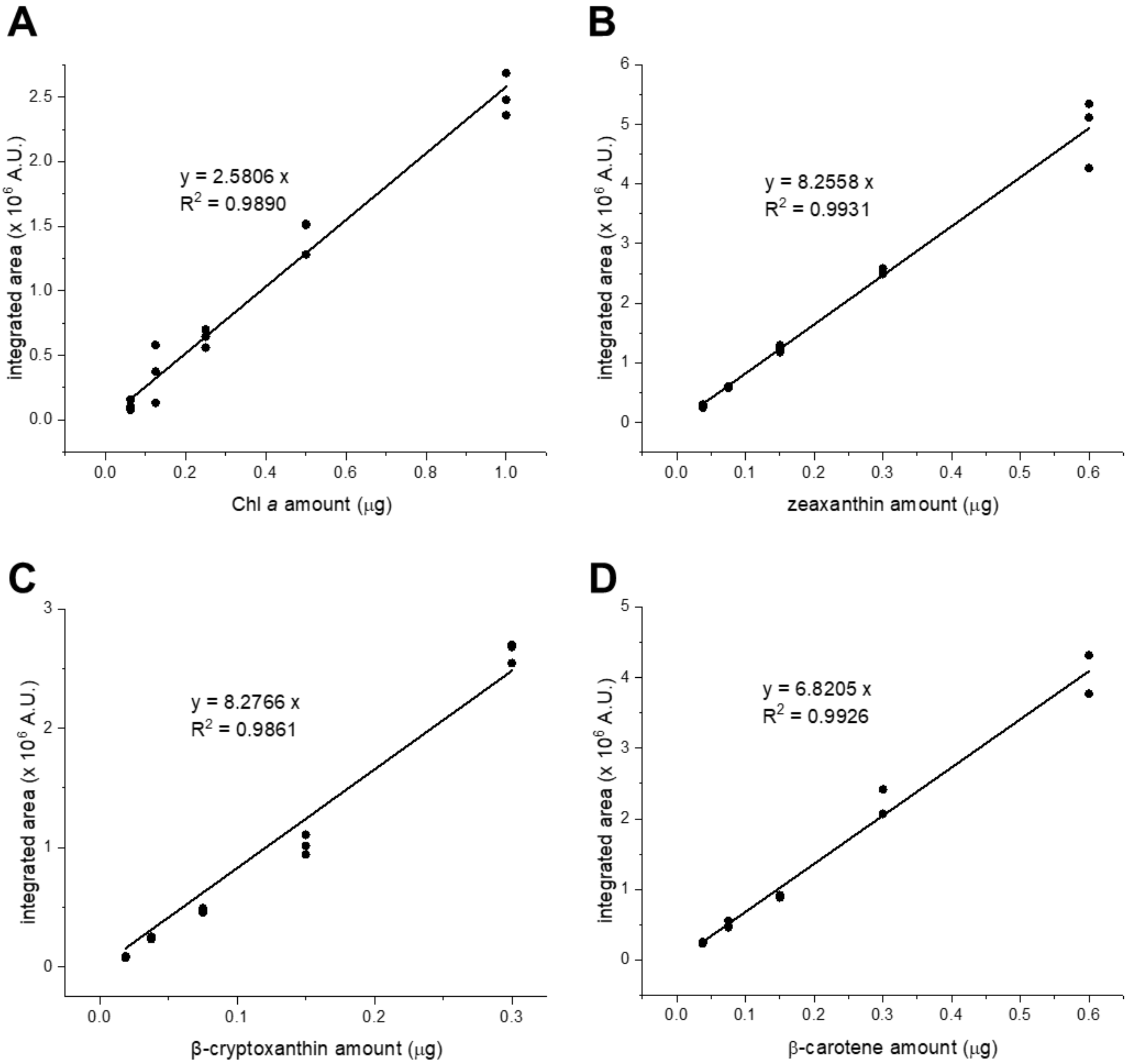

Supplementary Figure S2. Calibration curve of integrated area of the HPLC chromatogram detected at 436 nm and the concentration of Chl *a* (A), zeaxanthin (B), β-cryptoxanthin (C), and β-carotene (D).

Black dots denote the integrated area of five concentrations of indicated pigments in three replicates, and the black line indicates the calibration obtained through linear regression. The equation of the linear regression curve is used to estimate the amount of individual pigments.

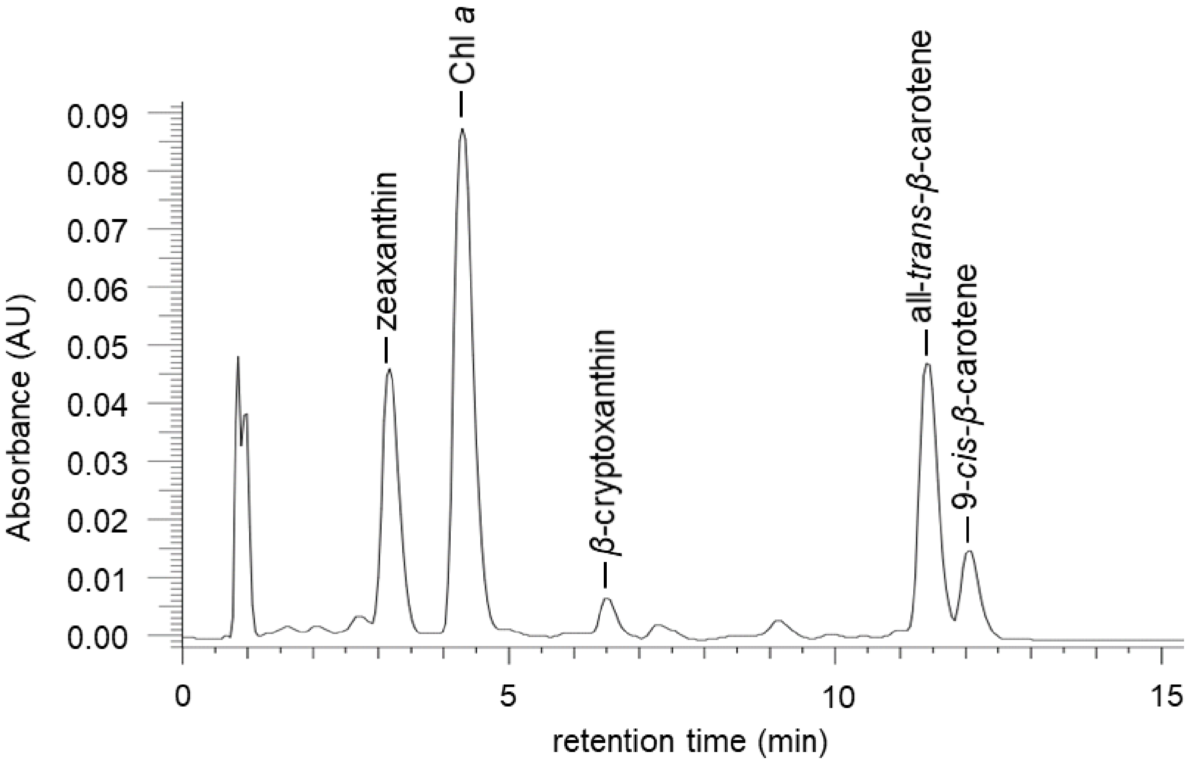

Supplementary Figure S3. Representative HPLC chromatogram of Chl and carotenoids in *G. partita*.

The elution condition for this HPLC chromatogram detected at 436 nm is a gradient of 0% to 30% mobile phase B (methanol:methyl tert-butyl ether:water = 7:90:3) in mobile phase A (methanol:methyl tert-butyl ether:water = 81:15:4) over 15 min. The peaks identical to those of authentic standard pigments are annotated.

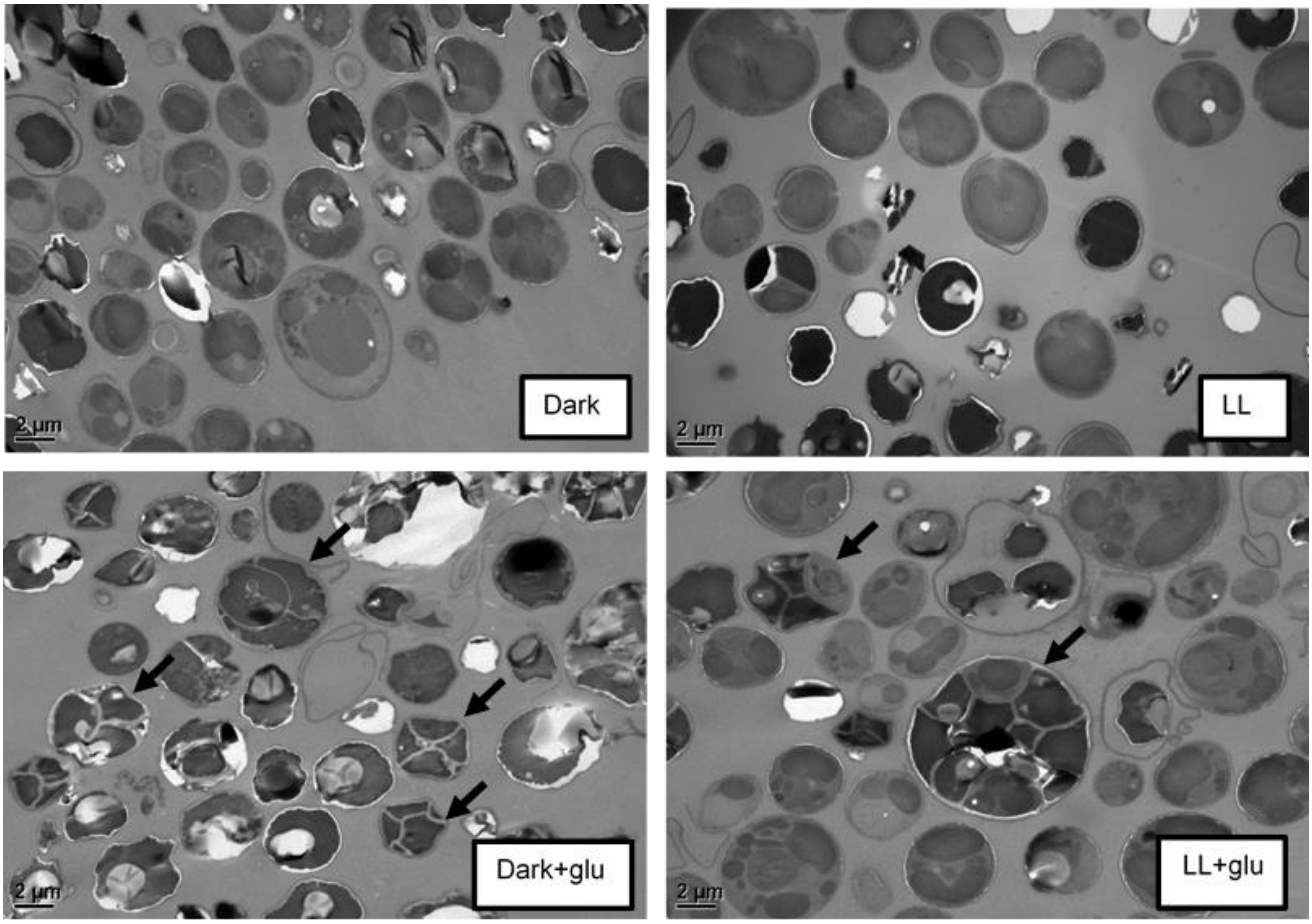

Supplementary Figure S4. Transmission electron micrographs showing various forms of *G. partita* cells under the dark, dark+glu, LL, and LL+glu conditions for 6 d.

Rapid cell proliferation is accompanied by a large proportion of multiple endospores (indicated by arrows) in the dark+glu and LL+glu conditions.

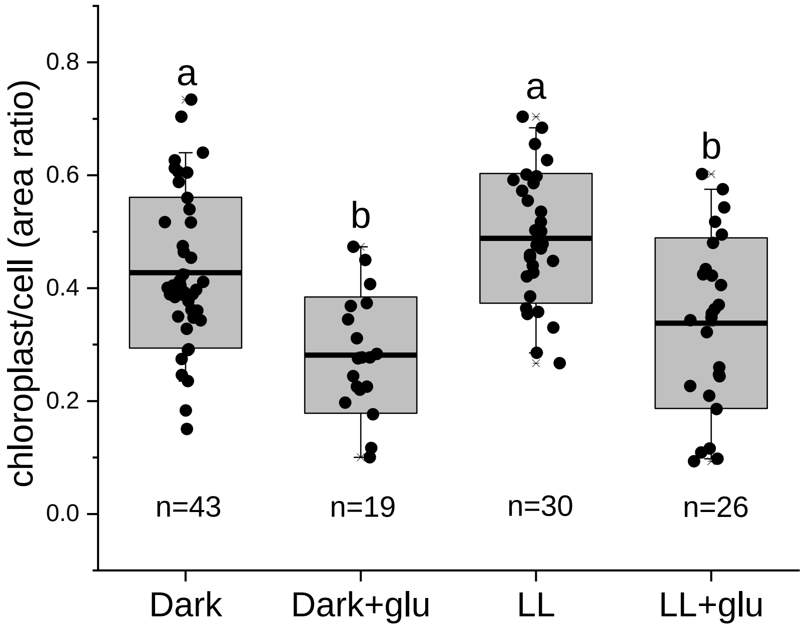

Supplementary Figure S5. Area ratio of chloroplast to cell indicating the size proportion of plastids in the *G. partita* cell under the dark, dark+glu, LL, and LL+glu conditions for 6 d.

The horizontal line denotes the mean value, and the box indicates mean ± SD with a whisker in the range of 5th–95th percentile. Black dots indicate the measured ratios of the indicated number of samples. Lowercase letters indicate significantly different groups as determined using one-way ANOVA followed by Tukey's multiple comparison test.

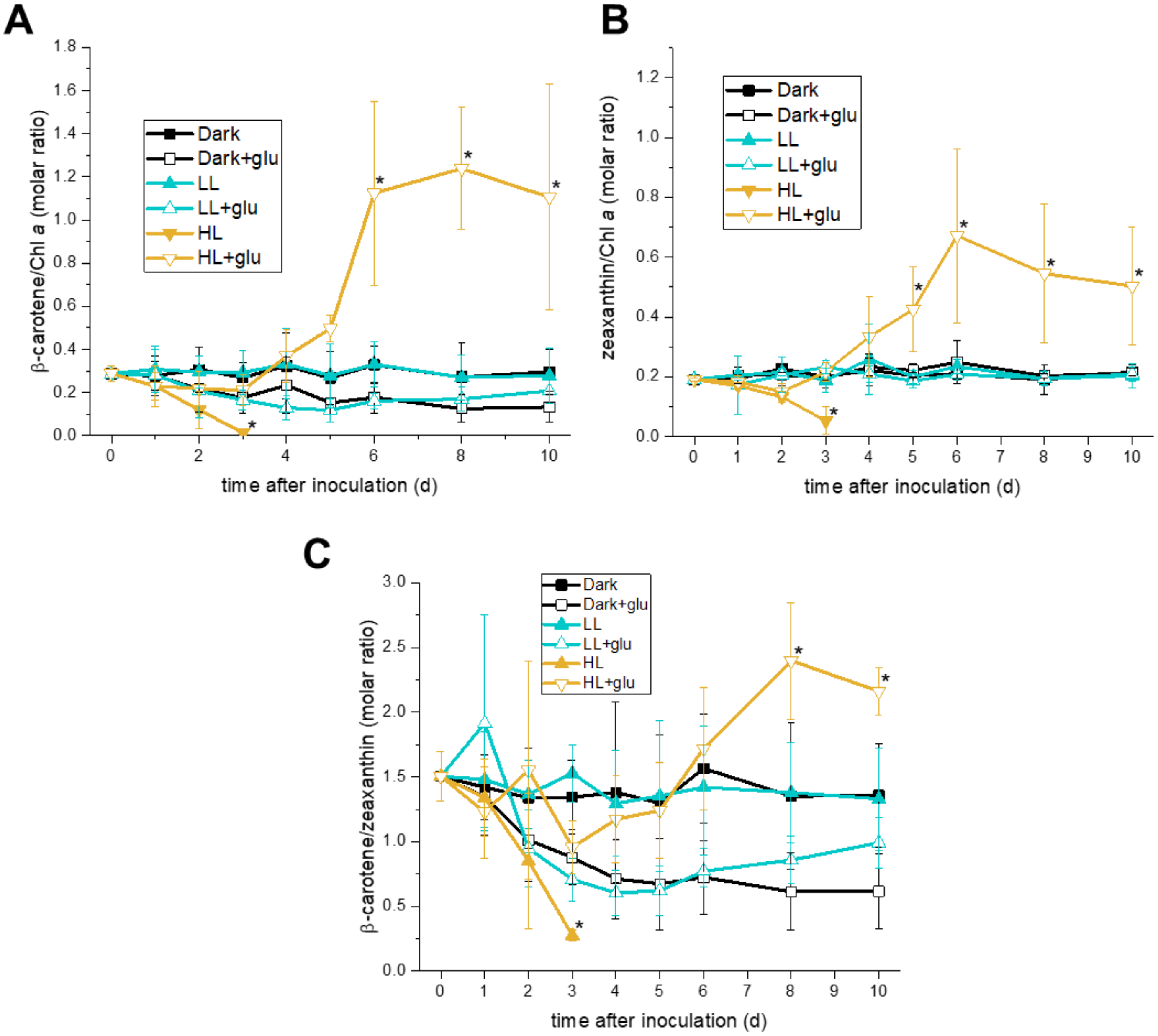

Supplementary Figure S6. Changes in the molar ratio of β-carotene/Chl *a* (A), zeaxanthin/Chl *a* (B), and β-carotene/zeaxanthin.

Cells were cultured in dark, LL, and HL conditions (equivalent to 0, 20, and 300 µmol photons m^{-2} s^{-1} PAR, respectively). The growth medium supplemented with 25 mM of glucose is denoted as '+glu'. Data are expressed as mean ± SD of three independent experiments. Asterisks indicate a significant difference from the other growth conditions on the same day as determined using one-way ANOVA followed by Tukey's multiple comparison test.

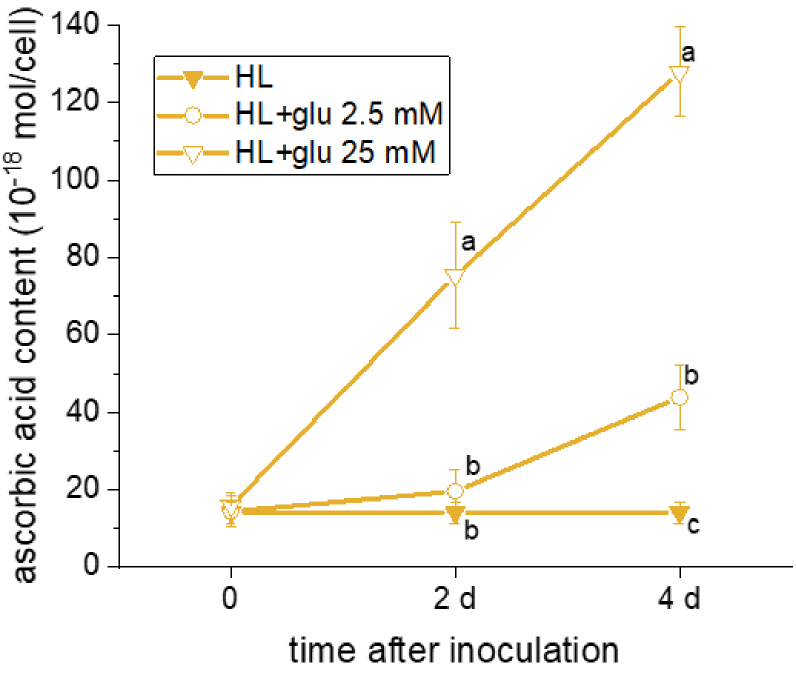

Supplementary Figure S7. Changes in the cellular content of ascorbic acid using the ascorbate oxidase-based assay kit (Abcam ab219928).

Cells were cultured supplemented of the indicated glucose concentrations under the HL condition (equivalent to 300 μmol photons m^{-2} s^{-1} PAR). Data are expressed as mean ± SD of three independent experiments. Lowercase letters indicate significantly different groups as determined using one-way ANOVA followed by Tukey's multiple comparison test.

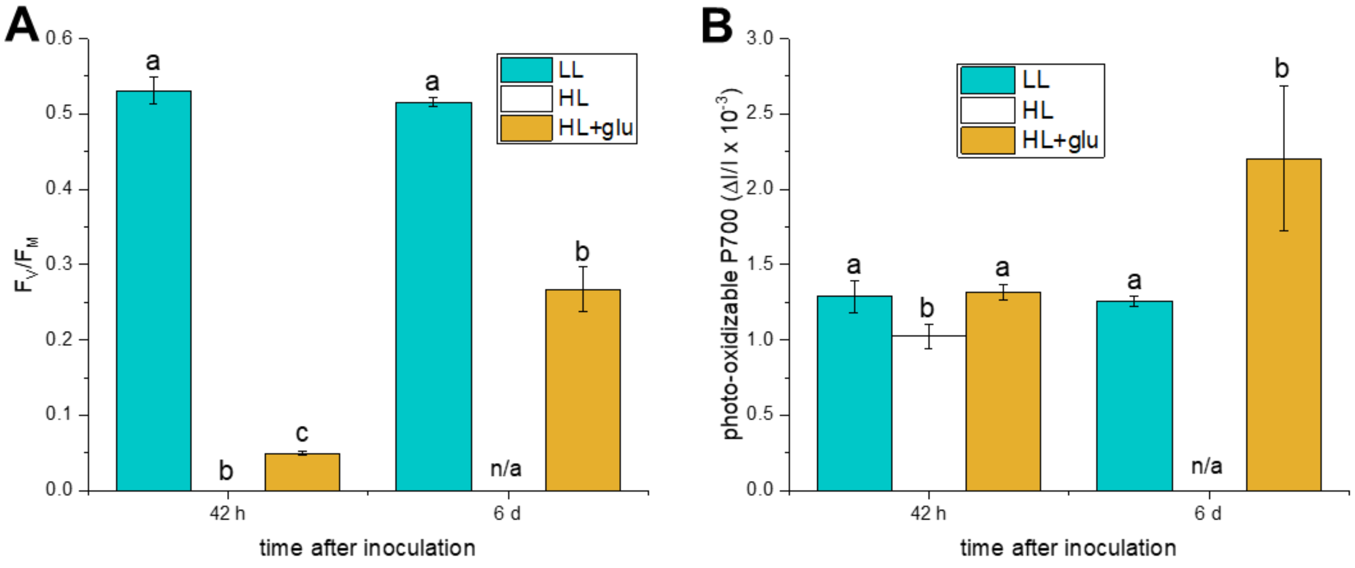

Supplementary Figure S8. Photoprotective response of supplemented glucose for 42 h and 6 d in the HL condition.

(A) F_V/F_M. (B) Photo-oxidizable P700 level determined at the same Chl *a* concentration. The values measured under the LL condition are used as reference without photoinhibition. Data are expressed as average ± SD of three independent experiments. Lowercase letters indicate significantly different groups as determined using one-way ANOVA followed by Tukey's multiple comparison test. n/a = not determined as cells perished.

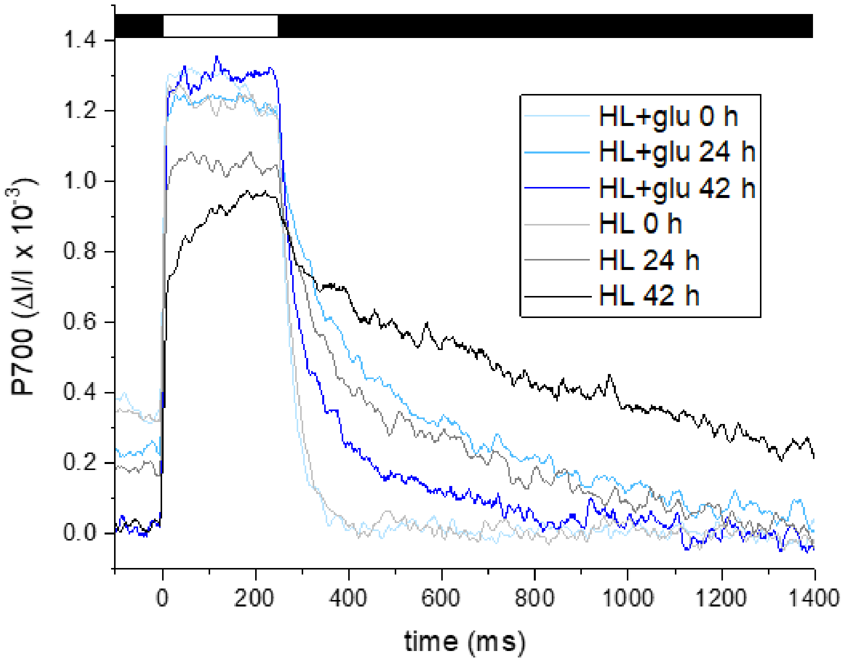

Supplementary Figure S9. Representative oxidation–reduction kinetic curves of P700 under the HL and HL+glu conditions for the indicated time.

P700 is oxidized to the maximum level during a 250-ms saturation pulse, as indicated by the white horizontal bar, and reduced after the pulse, as indicated by the black horizontal bar. The oxidation and reduction rates of P700 are considerably slow under the HL condition for 42 h, indicating severe inhibition of PSI activity.

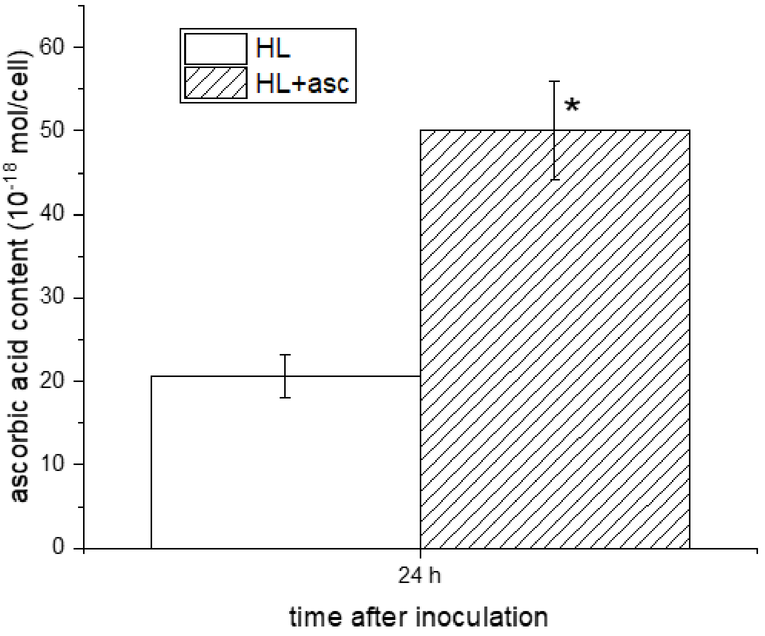

Supplementary Figure S10. Incorporation of ascorbic acid in the cell 1 d after inoculation.

The cellular content of ascorbic acid was estimated using the ascorbate oxidase-based assay kit (Abcam ab219928). Cells were cultured under the HL condition (equivalent to 300 µmol photons m^{-2} s^{-1} PAR), and the growth medium supplemented with 50 mM of ascorbic acid is denoted by '+asc'. Data are expressed as mean ± SD of three independent experiments. The asterisk indicates a significant difference from the HL condition as determined using a two-sample *t* test.

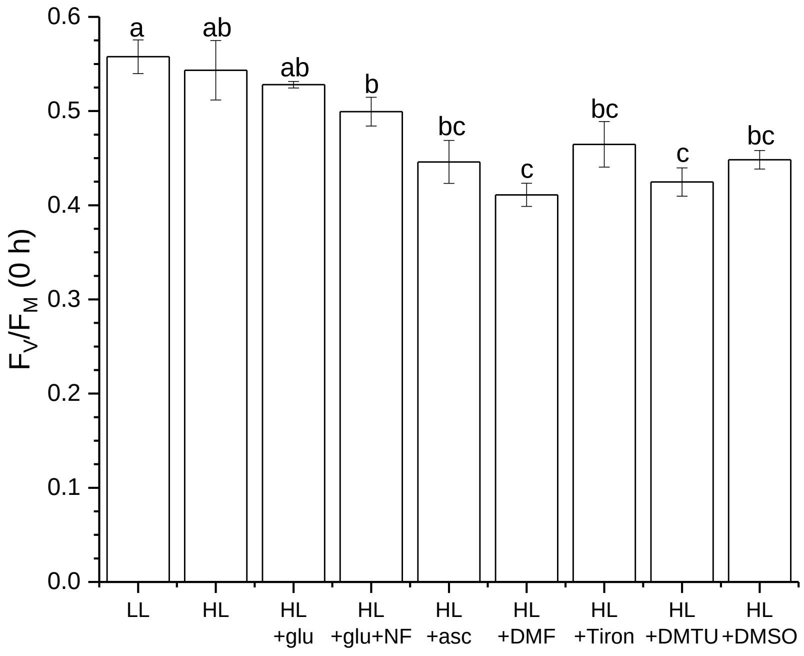

Supplementary Figure S11. F_V/F_M upon inoculation with ascorbic acid, norflurazon, or various ROS-specific scavengers.

Data are expressed as average ± SD of three independent experiments. Lowercase letters indicate significantly different groups as determined using one-way ANOVA followed by Tukey's multiple comparison test.

1.2 Supplementary Tables

Supplementary Table S1. Dissolved organic carbon (DOC) content and environmental parameters of soil and stream samples

Two soil samples and one stream sample were collected under a similar light regime on a cloudy day, March 28, 2018, at GengZiPing. The DOC content was expressed as the water extractable organic carbon (WEOC) content for the soil samples or the total organic carbon (TOC) content for the stream sample. WEOC was extracted from soil samples placed in conical flasks and filled with double distilled water. The flasks were sealed and shaken at 150 rpm for 60 min, and the soild samples were then filtered through the Whatman filter paper No.5 (2.5 µm). The DOC content of water extracts and the stream sample were analyzed with an OI-Analytical TOC analyzer (Model 1010, College Station, Texas, USA). Analyses were duplicated for each soil sample and tripleted for the stream sample. The pH, temperature, and photosynthetic active radiation (PAR) values were measured at the sampling sites before the samples were taken.

	Soil 1	Soil 2	Stream
DOC content [WEOC in soil (µg C/ g dry soil) or TOC in stream (µg C/ml)]	34.67	29.83	0.71
pH	0.4	0.4	2
Temperature (°C)	34.5	41.7	38.4
PAR (µmol photons m^{-2} s^{-1})	48	181	117

Supplementary Table S2. Cellular content of individual carotenoids under various growth conditions

Data are expressed as mean ± SD of three independent experiments. n.d. = not detectable.

Cellular content (10^{-18} mol/cell)		Day(s) after inoculation								
		0 d	1 d	2 d	3 d	4 d	5 d	6 d	8 d	10 d
zeaxanthin	Dark	34.50 ± 0.87	30.36 ± 2.03	36.10 ± 3.16	28.92 ± 2.04	33.56 ± 12.90	33.86 ± 1.61	30.98 ± 3.26	27.22 ± 4.03	31.89 ± 2.62
	Dark+glu		30.56 ± 8.77	21.82 ± 13.80	21.48 ± 11.08	30.83 ± 24.70	14.62 ± 4.28	14.79 ± 7.71	10.01 ± 3.18	9.72 ± 1.54
	LL		29.20 ± 1.73	39.19 ± 0.57	33.78 ± 4.76	53.70 ± 29.86	32.10 ± 2.34	37.31 ± 10.49	30.35 ± 4.83	32.73 ± 4.82
	LL+glu		22.31 ± 12.07	26.55 ± 10.27	19.29 ± 13.83	16.01 ± 4.86	15.66 ± 4.92	15.29 ± 7.27	10.90 ± 2.43	10.44 ± 2.70
	HL		20.59 ± 5.26	4.02 ± 2.24	0.27 ± 0.25	n.d.	n.d.	n.d.	n.d.	n.d.
	HL+glu		18.20 ± 6.25	4.92 ± 3.83	3.09 ± 2.73	2.11 ± 2.20	2.00 ± 2.03	5.19 ± 5.83	2.53 ± 1.27	1.55 ± 0.42
β-carotene	Dark	91.93 ± 8.24	77.96 ± 3.18	89.77 ± 7.77	72.50 ± 7.52	68.36 ± 26.42	83.11 ± 17.82	83.88 ± 6.07	68.31 ± 18.36	80.79 ± 12.79
	Dark+glu		77.01 ± 29.29	48.46 ± 35.66	41.66 ± 20.18	29.34 ± 3.74	24.98 ± 3.61	25.74 ± 11.01	16.57 ± 3.60	16.54 ± 0.66
	LL		77.26 ± 12.22	98.67 ± 11.00	92.11 ± 16.80	90.57 ± 25.50	79.44 ± 15.04	92.93 ± 11.78	75.61 ± 2.74	81.29 ± 15.95
	LL+glu		64.78 ± 31.49	60.62 ± 33.19	40.19 ± 13.27	28.52 ± 15.66	26.82 ± 4.34	31.02 ± 4.37	21.50 ± 3.76	21.64 ± 4.59
	HL		50.16 ± 10.19	7.46 ± 4.44	0.34 ± 0.31	n.d.	n.d.	n.d.	n.d.	n.d.
	HL+glu		48.56 ± 18.77	16.85 ± 9.39	8.75 ± 5.40	6.31 ± 4.91	5.85 ± 4.22	18.34 ± 15.19	10.39 ± 5.87	5.86 ± 0.86
β-cryptoxanthin	Dark	4.76 ± 0.36	4.76 ± 0.36	6.05 ± 0.17	4.93 ± 0.41	6.09 ± 2.20	5.61 ± 1.81	5.21 ± 0.29	4.11 ± 0.69	5.88 ± 0.83
	Dark+glu		4.14 ± 1.12	2.44 ± 1.54	2.48 ± 1.88	4.72 ± 5.60	1.34 ± 0.16	1.21 ± 0.42	0.93 ± 0.37	1.09 ± 0.35
	LL		4.78 ± 0.22	5.95 ± 0.40	6.44 ± 1.42	8.39 ± 4.45	4.59 ± 0.79	5.88 ± 1.00	4.58 ± 0.10	5.16 ± 0.86
	LL+glu		3.32 ± 1.44	2.75 ± 1.03	1.78 ± 1.61	1.24 ± 0.22	1.28 ± 0.35	1.71 ± 1.02	1.15 ± 0.37	1.25 ± 0.38
	HL		2.76 ± 0.89	0.22 ± 0.10	n.d.	n.d.	n.d.	n.d.	n.d.	n.d.
	HL+glu		2.15 ± 1.01	0.54 ± 0.43	0.39 ± 0.45	0.51 ± 0.56	0.45 ± 0.38	1.32 ± 1.43	0.75 ± 0.30	0.48 ± 0.09
